# Annotations

**Published:** 1905-07-15

**Authors:** 


### ANNOTATIONS.

### Mr. Tweedy on the Medical Curriculum.

In an address on the occasion of the distribution of prizes at the medical school of the Westminster Hospital, the President of the Royal College of Surgeons took the opportunity to congratulate the authorities on the arrangement under which the students of the school will in future pursue their preliminary scientific studies, not in the medical school, but at King's College. The teaching of such subjects in medical schools connected with hospitals, he considered, had for some time stood in the way of the best medical education. The great value of chemistry, physics and biology to the student of medicine was found in the fact that these studies afforded an education in scientific methods, and such methods, and the discipline of the mind they afford, are best obtained in institutions devoted to the teaching of pure science. Mr. Tweedy had no doubt that the adoption of the scheme would be in the interests of the individual student and also favourable to the progress of medical education. Another scheme to be tested at the Westminster medical school is the division of the courses of lectures on systematic medicine and systematic surgery among a number of teachers. That is to say, instead of one teacher lecturing, for example, on medicine, there will be individual teachers for different groups of diseases. This experiment will have to be carefully watched, and it is far from certain that its influence will be a wholesome one. Obviously it must extend and emphasise the development of specialism, and must, to a corresponding extent, weaken the cultivation of that general and comprehensive view on which the older physicians and surgeons ever insisted. Mr. Tweedy's suggestion that in addition to the specialised lectures there should be a sort of propædeutic or introductory course expounding the great general principles of medicine and surgery is an excellent one, and we hope it will not be neglected. The address concluded with some sound remarks on the intimate relationship between clinical study and observation, and the actual practice of medicine and surgery. It is in the wards of the hospital that the student finds the opportunities for the construction of the foundation on which he is to build the work of his life.

### Damages for Traumatic Hysteria.

A case of considerable medico-legal interest has recently been decided in the Canadian High Court of Justice. It is probably the first instance on record in which compensation has been awarded by legal process for a condition induced by a railway accident and admitted to be one of pure hysteria. The plaintiff was a young woman, twenty-five years of age, and by profession an artist. When travelling by an electric railway something of the nature of an explosion occurred, and the plaintiff either jumped or fell off the car. There was little or no evidence of physical injury, but in the course of some hours she became unconscious, and remained in this condition for several days. Afterwards she was found to be paralysed on the left side, the paralysis being accompanied by left hemi-anæsthesia. These symptoms had persisted for ten months, during the course of which there were several attacks marked by violent epileptiform convulsions. It was admitted by all the medical witnesses that the diagnosis of traumatic hysteria was correct, and the Court ruled that the term "injury" must be held to include fright as well as shock and physical injury. The prognosis in reference to the probable date of recovery gave rise, not unnaturally, to considerable difference of opinion, but it was generally allowed that the termination of legal proceedings would tend to expedite recovery. In the end, the jury awarded the plaintiff the sum of 10,000 dols. It is, we think, a decided gain that a court of justice and a jury can have been educated to appreciate the fact that a functional disturbance is a real injury and not a mere sham and pretence on the part of the sufferer. The distinction, though a very essential one, does not readily penetrate the lay mind, and the precedent now created may be of value in dealing with this very difficult class of cases. Even members of the medical profession sometimes need to be reminded that functional paralysis and allied conditions undoubtedly exist. Such cases are not infrequently interpreted as the result of a definite organic lesion, and thus prognosis and treatment are directed on to essentially wrong lines. When once the correct diagnosis is reached, the difficulty is to persuade the patient's friends and other interested persons that the case is not one of malingering. This difficulty has been successfully overcome at Toronto, and it will be of interest to note whether a British jury, when the occasion arises, will be equally open to argument.

### Protection for Medical Practitioners.

It is true that the two Bills which Sir John Batty Tuke has prepared, with the approval of the General Medical Council and in co-operation with well-known medical men, for the protection of practitioners in medicine and dentistry, have the cordial goodwill of the President and Secretary of the Board of Trade. But the cordial goodwill of Ministers is not sufficient to compass legislation at the fag end of the Session, and we shall be pleasantly surprised if the member for Edinburgh and St. Andrews Universities is able to get his two measures added to the Statute Book. Yet the necessity for their enactment is so pressing, and their object is so unimpeachable, that they ought not to share the fate of highly controversial schemes. The evils of allowing joint stock companies either to act as physicians or surgeons, or to pursue the practice of dentistry, must be obvious. It is only necessary, in the existing state of affairs, to comply with the formalities of the Joint Stock Companies Act in order to drive a coach and four through the Medical and Dentists Acts. Over and over again this has been done, and it is well known that men innocent of all legal and proper qualifications manage to practise both medicine and dentistry under the cover of a company registration. Both on account of the public, who are misled by advertising methods, and in justice to duly qualified medical men and dentists, there is urgent need for action. No one but persons directly interested in the spurious combinations to which exception is here taken, can object to legislation intended to prevent the profession of medical and dental practice except by persons duly qualified.

